# Emotional Content and Source Memory for Language: Impairment in an Incidental Encoding Task

**DOI:** 10.3389/fpsyg.2019.00065

**Published:** 2019-01-30

**Authors:** Pilar Ferré, Montserrat Comesaña, Marc Guasch

**Affiliations:** ^1^Department of Psychology and CRAMC, Universitat Rovira i Virgili, Tarragona, Spain; ^2^Human Cognition Lab, Research Center on Psychology, School of Psychology, University of Minho, Braga, Portugal

**Keywords:** source memory, emotional words, language, bilingualism, intrinsic source

## Abstract

Research into the effects of emotion on source memory (i.e., memory for certain contextual details of a stimulus, such as its location, color, or temporal context) has yielded inconsistent findings. Mather and her co-workers tried to account for such inconsistencies by pointing out the relevance of the characteristics of the feature examined. Specifically, they distinguished between intrinsic and extrinsic features (Mather, 2007) and between goal-relevant and goal-irrelevant information (Mather and Sutherland, 2011). In the current study, we investigated source memory for language, which is an intrinsic feature or words. Catalan-Spanish bilinguals were tested in three experiments involving a recognition task in which they were asked about the language of presentation (Catalan or Spanish) of emotional and neutral words. In Experiments 1 and 2, source memory for negative and neutral words was assessed. In Experiment 1 participants performed an intentional encoding task in which language was a goal-relevant feature. In Experiment 2, they did an incidental encoding task in which language was also goal-relevant. Experiment 3 replicated Experiment 2 but negative words were replaced by positive words. The results showed an impairment in source memory for the language of presentation of emotional words when the encoding task was incidental, but not when it was intentional. Such impairment was observed with both negative words and positive words. The results are discussed in relation to the proposals of Mather and co-workers and point to the relevance of modulating factors, such as the intentional/incidental nature of encoding.

## Introduction

The last decades have witnessed a great interest in the study of the effects of emotion on episodic memory. The majority of the studies in the field have been concerned with item memory (i.e., memory for the content of the information), finding that emotional stimuli are better remembered than neutral stimuli (see LaBar and Cabeza, 2006; Mather and Sutherland, 2011; Phelps, 2012; Dolcos et al., 2017; Kensinger and Kark, 2018; for overviews). However, far less research has been devoted to the effects of emotion on source memory (see Chiu et al., 2013, for a review). Source memory refers to memory for certain contextual details of an event (Johnson et al., 1993). It is commonly assessed using recognition memory tasks in which participants are not only asked to recognize previously presented information as old, but are also required to retrieve encoding details about such information. Studies in this field have compared emotional stimuli (defined in terms of their valence and arousal levels) and neutral stimuli (commonly images or words) and have tested memory for different types of information, such as temporal or spatial information, cognitive operations performed during encoding, or visual-perceptual stimulus features, among others (Chiu et al., 2013). In all these studies, the amount of source information is much lower than the number of trials, as responses involve a limited number of alternative choices (e.g., participants have to decide between two colors, or two different spatial locations). A related line of research has focused on memory for relational/associative information, where there is a one-to-one relationship between trials and the relational information (e.g., participants have to remember the background scenes presented together with emotional/neutral items or the two items which were presented in pairs during encoding, see Chiu et al., 2013, for a detailed description). Although the terms “source memory” and “relational/associative memory” do not refer exactly to the same, the main findings of both lines of research have to be taken into consideration in order to have a complete picture of the field. Of note, the present study fails within the first group of studies and sought to test the effects of emotion on source memory for language.

Research into the effects of emotion on source memory and relational memory has yielded mixed findings: a number of studies have shown that emotional content enhances memory for location (D’Argembeau and Van der Linden, 2004; Mather and Nesmith, 2008; Nashiro and Mather, 2011; Schmidt et al., 2011; Rimmele et al., 2012), color (Doerksen and Shimamura, 2001; Kensinger and Corkin, 2003), temporal information (D’Argembeau and Van der Linden, 2005; Schmidt et al., 2011; Rimmele et al., 2012), the task performed with the items at encoding (Kensinger and Schacter, 2006), or the identity of objects associated to background scenes (Smith et al., 2005). This enhanced memory was associated, in the last study, with differential neural activity during the retrieval of information associated with emotional background scenes. A number of studies, however, have found a different pattern: an impairment of emotional stimuli in the memory for location (Mather et al., 2006; Mitchell et al., 2006; Mather and Knight, 2008; Maddock and Frein, 2009), color (Mackenzie et al., 2015) and the type of task performed during encoding (Cook et al., 2007; Mao et al., 2015). Such impairment was also observed at a neural level, where event-related potential (ERP) correlates of source memory were observed for neutral items, but not for emotional ones (Mao et al., 2015). Similarly, a series of studies have reported an impairment in memory for the peripheral information of scenes containing a central emotional item (e.g., Kensinger et al., 2007; Bisby and Burgess, 2014) as well as for items associated with emotional stimuli in paired designs (e.g., Mather and Knight, 2008; Nashiro and Mather, 2011; Pierce and Kensinger, 2011; Bisby and Burgess, 2014). Apart from these contrasting findings, other studies have failed to find any effect of emotion on source memory and relational memory at all (e.g., Sharot and Yonelinas, 2008; Mather et al., 2009; Meyer et al., 2015).

The reason of the inconsistent pattern of findings above reviewed is far from being established. However, they suggest that several factors can be modulating emotion effects. Mather and her colleagues have highlighted the role of certain aspects of the information. In her first proposal, the object-based framework, Mather (2007) argued that the crucial distinction was between intrinsic information (i.e., features that are an integral part of an item, such as its color) and extrinsic information (i.e., features that are not an integral part of an item, such as the context of an emotional scene). According to Mather, emotional (arousing) information would attract attention, enhancing item-context binding only when the context belongs to the same object as the emotional information (i.e., intrinsic information). This proposal has been supported both by studies reporting a memory enhancement with intrinsic information, such as color or location (Doerksen and Shimamura, 2001; D’Argembeau and Van der Linden, 2005; Mackay and Ahmetzanov, 2005; Mather and Nesmith, 2008; Nashiro and Mather, 2011) and by those reporting a memory impairment with peripheral details of scenes (e.g., Kensinger et al., 2007) or the binding between pairs of items (e.g., Nashiro and Mather, 2011; Pierce and Kensinger, 2011). However, the inconsistencies in the literature cannot be entirely resolved by Mather’s account. For instance, both null effects (Meyer et al., 2015) and detrimental effects (Mackenzie et al., 2015) of emotion on intrinsic features of the stimuli have recently been reported. Even more, the same type of information (e.g., location, an intrinsic feature) has shown an enhancement in some studies (D’Argembeau and Van der Linden, 2004; Mackay and Ahmetzanov, 2005) and an impairment in others (Mather et al., 2006; Maddock and Frein, 2009).

In light of the above, Mather and co-workers updated and extended their model by emphasizing the role of a different factor, namely stimulus priority. Given limited human attention capacity, it is necessary to prioritize one piece of information above others during processing. In the arousal-biased competition (ABC) theory, Mather and Sutherland (2011) argued that emotional arousal would have a role in such prioritization, by enhancing processing (and memory) of the highest priority information, while impairing processing (and memory) of the lowest priority information. Therefore, the ABC theory predicts an emotion enhancement in memory for high priority information, regardless of its intrinsic/extrinsic nature. In this way, it can account for previous evidence of emotion improvement in memory for what can be considered extrinsic sources of information. For instance, Guillet and Arndt (2009) reported enhanced memory for neutral words associated with emotional words. Of note, in this study participants were asked to learn word-word associations, thus making the associated neutral words become priority information. Similarly, instructing participants to intentionally connect the location of objects with background scenes led to an enhancement in memory for the identity of objects (Ventura-Bort et al., 2016) and for their location (Luck et al., 2014) when the background scenes were emotional. Interestingly, this effect was associated with an enhancement of one ERP indicator of recognition memory (the old/new effect, Ventura-Bort et al., 2016). It was also associated with increased activity in the amygdala during encoding (Luck et al., 2014), suggesting that the interaction between this structure and medial temporal lobe regions involved in memory binding might be responsible for the superior memory for goal-relevant information (Dolcos et al., 2017).

The majority of the studies reviewed in the above paragraphs (mainly those conducted before 2012) were not carried out to test the ABC theory. However, we can examine the extent to which the features examined were goal-relevant (i.e., relevant for the task to be performed) by looking at the encoding instructions. In doing so, the ABC does not seem to be entirely supported. In particular, although the enhancing emotion effects in memory for information that was relevant for a specific task have been reported (e.g., Doerksen and Shimamura, 2001), both null effects (Meyer et al., 2015) and detrimental effects (Maddock and Frein, 2009; Otani et al., 2012; Mackenzie et al., 2015) have also been obtained. On the contrary, enhancing effects have been reported with information which was not at all relevant during encoding (e.g., Nashiro and Mather, 2011; Schmidt et al., 2011; Rimmele et al., 2012).

Overall, it seems that the proposals of Mather and co-workers cannot account entirely for the discrepant findings in the field. A possible reason may be that other factors, such as the valence of the stimuli or the nature of the encoding task, can modulate the pattern of effects. Regarding valence, although some studies have reported an effect of emotion on source and relational memory for both negative and positive information (e.g., Mather and Nesmith, 2008; Schmidt et al., 2011; Mao et al., 2015), a number of studies have failed to find any effect with positive stimuli (e.g., D’Argembeau and Van der Linden, 2005; Cook et al., 2007; Maddock and Frein, 2009; Otani et al., 2012). These results suggest that the attraction of attention that can ultimately lead to an effect on source and relational memory seems to affect mainly negative information. With respect to the nature of the encoding task, some studies have relied on intentional encoding tasks, in which participants are encouraged to memorize both the item and the source (Otani et al., 2012; Mackenzie et al., 2015; Mao et al., 2015), or only the item (Cook et al., 2007). Other studies have relied on incidental tasks in which participants are not told about any subsequent memory test (Maddock and Frein, 2009; Nashiro and Mather, 2011; Schmidt et al., 2011; Bisby and Burgess, 2014). Arguably, the intention (vs. the lack of intention) to memorize both the content and the context of given information may affect the extent to which emotion affects memory of the context. In particular, if the influence of emotion on cognition involves the automatic activation of attention (D’Argembeau and Van der Linden, 2004), the effects of emotion on source memory might be more easily observed when participants do not intentionally memorize the source.

In the present study, we aimed to test the effects of emotion on source memory by using as source information a feature never before studied in the field: the language in which words were presented. Psycholinguistic research has demonstrated that there are distinct levels of word representation. Some of these have to do with the form of the word itself (i.e., orthography, phonology) and others with its meaning (e.g., Carreiras et al., 2014). When people know more than one language (i.e., they are bilinguals), each word in their lexicon must be identified in relation to the language to which it belongs. Therefore, language is part of word representation (Dijkstra and van Heuven, 2002). Since the meaning of words can include emotional connotations and their forms contain information about language, language constitutes a good means of testing the effect of emotion on source memory. Importantly, what makes language a feature worth to be investigated in relation to source memory is that it is highly integrated with item information, being clearly an intrinsic property of the words (see Bar-Elli, 2006).

Catalan-Spanish bilinguals were tested in this study in three memory recognition experiments in which Catalan and Spanish words were presented during the acquisition phase. Both valence and the type of encoding task (intentional vs. incidental) were manipulated. In Experiment 1, source memory for negative and neutral words intentionally encoded was assessed, language being a goal-relevant feature (i.e., participants were told to encode both the words and their language of presentation during the acquisition phase). In Experiment 2, the encoding task was incidental, although language was also a relevant feature (i.e., participants were asked to name the words in the correct language, without being informed that their memory of the words or the language would be tested afterward). Experiment 3 replicated Experiment 2, but negative words were replaced with positive ones in an attempt to evaluate the impact of valence on source memory. This manipulation was only done in the incidental task because the effects of emotion on memory were restricted to this type of codification, as will be described below. Considering that language was an intrinsic and goal-relevant feature in the three experiments, we might expect an improvement in memory for the language of presentation of emotional words in comparison to neutral words in all of them. However, modulations in the effect of emotion on source memory by the type of encoding task and the valence of the stimuli might be also expected if we bear in mind the literature here (Cook et al., 2007; Mather, 2007; Otani et al., 2012). In particular, we would expect a stronger effect of emotion on source memory in the incidental encoding task and with negative words.

## Experiment 1

This experiment was carried out in accordance with the code of ethics of the World Medical Association (Declaration of Helsinki) and was approved by the Ethics Committee for Human Research (SECH 017/2015) of the Research Center on Psychology (CIPsi) at the University of Minho. Furthermore, participants gave written informed consent.

### Methods

#### Participants

Thirty undergraduate students (23 females) from the University Rovira i Virgili (Tarragona, Spain) took part in the experiment either voluntarily or in exchange of academic credits. Their mean age was 22.27 years (*SD* = 4.36). All the participants had normal vision or corrected-to-normal vision and were bilinguals of Catalan and Spanish. Of note, the degree of bilingualism in Catalonia is very high. People living there acquire Catalan and Spanish in early childhood and are immersed throughout their lives in both languages. Hence, all the participants here were highly proficient early bilinguals of Catalan and Spanish, living in Catalonia at the time of the experiment, and using both Catalan and Spanish on a regular basis.

Participants were asked to fulfill a language history questionnaire to assess their degree of bilingualism. Data from the questionnaire confirmed that they were highly proficient in both languages. The average proficiency was 6.77 (*SD* = 0.39, Minimum = 5.75, Maximum = 7.00, Range = 1.25) for Catalan and 6.85 (*SD* = 0.30, Minimum = 5.75, Maximum = 7.00, Range = 1.25) for Spanish on a seven-point scale (being 1 a ‘very poor level,’ and 7 a ‘very good level’). The difference in self-rated proficiency between both languages was not significant; *t*(29) = 1.14, *p* = 0.265. Additionally, participants rated their frequency of language use on a seven-point scale (were 1 was ‘only Catalan’ and 7 was ‘only Spanish’), their mean being 4.02 (*SD* = 0.88, Minimum = 2.25, Maximum = 6.00, Range = 3.75). Finally, participants also rated their language preference using the same scale (i.e., 1 = ‘only Catalan’; 7 = ‘only Spanish’), the average rating being 4.18 (*SD* = 0.91, Minimum = 1.75, Maximum = 6.50, Range = 4.75). We carried out one-sampled *t*-tests to examine whether frequency and preference ratings were significantly different from the central point of the scale (i.e., ‘to the same extent in Catalan and Spanish’). These analyses failed to reveal a significant difference (both *p*s > 0.25), indicating that participants preferred and used both languages to the same extent.

#### Design and Materials

The experiment employed a 2x2 design. The factors involved were language (words presented in Catalan vs. Spanish) and emotion (negative vs. neutral words). The critical stimuli consisted of a set of 60 Spanish words and their Catalan translations (see Appendix). These were the words to be learned during the acquisition phase (i.e., old words). They were obtained from several Spanish normative databases, using the emoFinder online search engine (Fraga et al., 2018). Values were taken mainly from the Spanish adaptation of ANEW (Redondo et al., 2007), but some values were not available here and were obtained from Ferré et al. (2012), Guasch et al. (2016), and Hinojosa et al. (2016). Half the stimuli where negative words (e.g., *mentira* in Spanish and *mentida* in Catalan, meaning ‘lie’ in English) and the other half were neutral words (e.g., *sendero* in Spanish and *sender* in Catalan, meaning ‘path’ in English). A word was considered negative if it had a value of valence below 4 on a 9-point scale (1 = ‘very unpleasant,’ 9 = ‘very pleasant’), as well as a value of arousal above 5 on a 9-point scale (1 = ‘completely calm,’ 9 = ‘completely excited’). On the other hand, a word was considered neutral if it had a value of valence between 4 and 6, as well as a value of arousal below 5. Of note, we relied on affective ratings for Spanish words, since there are no normative affective ratings available for Catalan words. We assumed that the affective charge is the same for words in the two languages. The reasons for this are twofold: on the one hand, all the experimental words were cognates in the two languages (i.e., translation equivalents that are very similar in form, e.g., *cicatriz-cicatriu*, scar). On the other hand, the results of previous studies carried out with highly proficient early bilinguals of Catalan and Spanish (i.e., like those tested in this study) involving emotional and neutral words have repeatedly failed to find any interaction between the emotion effects and language (Ferré et al., 2010, 2013, 2018).

Negative and neutral words were matched on several psycholinguistic variables: length of the Catalan and the Spanish words, lexical frequency of the Catalan and the Spanish words, concreteness, imageability, subjective familiarity, and age of acquisition (AoA). The frequency of the Catalan words (i.e., word frequency per million) was obtained from the IEC (Rafel, 1998) through the online tool NIM (Guasch et al., 2013). The frequency of the Spanish words (also word frequency per million) was obtained from the subtitle corpus of EsPal (Duchon et al., 2013). EsPal was also the source of the subjective ratings of concreteness, imageability, and familiarity (with the exception of some unavailable data, which were obtained from Guasch et al., 2016). Age of acquisition was taken from Alonso et al. (2015). Of note, as above explained, all the critical stimuli were cognate words in Catalan and Spanish. The reason for this was to make the source memory task more difficult for participants. We reasoned that it would be too easy to remember the language in which words were presented if the form of the translation equivalents was different between the languages, the so-called non-cognate words (e.g., *estiu*-*verano*, ‘summer’). Notably, we excluded identical cognate translations in Catalan and Spanish (e.g., *amor*-*amor*, ‘love’) because this kind of stimuli would not have allowed participants to discriminate between the two languages. Hence, we matched the degree of orthographic similarity between translation equivalents across experimental conditions by computing the normalized Levenshtein distance (NLD) as described by Schepens et al. (2012). To that end, we used the online tool NIM (Guasch et al., 2013). Independent samples *t*-tests were used to check that the experimental conditions differed only in the manipulated variables. These tests showed significant differences between negative words and neutral words both in valence, *t*(58) = 19.80, *p* < 0.001, and arousal, *t*(58) = 14.34, *p* < 0.001. There were not significant differences in any of the controlled variables (all *p*s > 0.257). Table 1 shows the affective, semantic and lexical characteristics of the stimuli.

**Table 1:** Affective, semantic and lexical characteristics of the stimuli (old and new words) used in Experiments 1–3 (standard deviations in parentheses).

	“Old” words			“New” words		
	Negative	Neutral	Positive	Negative	Neutral	Positive
Valence	2.24 (0.55)	5.13 (0.58)	7.46 (0.57)	2.21 (0.66)	5.11 (0.48)	7.48 (0.59)
Arousal	6.53 (0.76)	4.13 (0.52)	6.02 (0.57)	6.56 (0.88)	4.08 (0.46)	6.17 (0.77)
Spanish frequency	14.84 (19.26)	11.32 (15.44)	14.66 (15.23)	8.73 (9.11)	9.51 (9.27)	13.68 (26.37)
Catalan frequency	11.96 (12.42)	16.68 (18.87)	27.04 (31.55)	12.21 (14.69)	15.35 (11.84)	20.90 (21.53)
Spanish length	8.37 (1.81)	8.07 (1.60)	8.57 (2.03)	8.53 (1.76)	8.47 (1.81)	9.10 (1.83)
Catalan length	7.80 (1.90)	7.47 (1.70)	8.13 (2.11)	7.83 (1.66)	7.80 (1.85)	8.57 (2.01)
NLD	0.84 (0.07)	0.83 (0.07)	0.84 (0.07)	0.85 (0.06)	0.85 (0.06)	0.84 (0.06)
Concreteness	4.63 (0.97)	4.71 (1.09)	4.33 (0.72)	4.56 (0.85)	4.43 (1.07)	4.12 (0.88)
Imageability	4.61 (0.90)	4.79 (1.20)	4.65 (1.11)	4.50 (1.07)	4.22 (1.33)	4.17 (1.26)
Familiarity	5.22 (0.85)	5.28 (0.93)	5.52 (0.64)	4.87 (0.83)	4.92 (0.77)	5.19 (0.49)
AoA	7.85 (1.64)	7.79 (1.57)	7.42 (1.71)	8.20 (1.58)	8.29 (1.74)	7.55 (1.78)

We created two experimental lists. In each list, half the words appeared in Spanish and the other half appeared in Catalan. The language of presentation was counterbalanced across lists. Hence, any word that appeared in Spanish in one list, appeared in Catalan in the other list, and *vice versa*.

Finally, in addition to the 60 words to be learned, we selected another set of 60 words to be used in the test phase of the recognition task as new words. These items were selected according to the same criteria as the critical items, and followed the same distribution in experimental conditions. The statistical analyses showed that there were clear differences between negative and neutral new words in both valence, *t*(58) = 19.51, *p* < 0.001, and arousal, *t*(58) = 13.64, *p* < 0.001. There were no differences in any of the above mentioned controlled variables (all *p*s > 0.366). Furthermore, *t*-tests carried out between old and new negative words, and between old and new neutral words, confirmed that the old and new sets of words were correctly matched (all *ps* > 0.09).

#### Procedure

Participants performed a recognition memory task consisting of three phases. The first of these was the acquisition phase, followed by a distractor phase, and the test phase.

During the acquisition phase, participants had to memorize a list of words presented on the screen. Those words could appear either in Catalan or in Spanish. Participants knew that their memory of the words would be tested afterward. They were encouraged not only to memorize the words, but also the language in which they appeared. Participants were presented with a set of 60 words belonging to the critical conditions (i.e., 15 Spanish negative words, 15 Spanish neutral words, 15 Catalan negative words, and 15 Catalan neutral words). We created two experimental files. The language of presentation was counterbalanced across files. Hence, any word that appeared in Spanish in one file, appeared in Catalan in the other file, and *vice versa*. Apart from the 60 critical items, each file included 8 filler items: 4 at the beginning and 4 at the end. These words were included to control for serial position effects (primacy and recency effects, respectively) and had the same characteristics as the experimental words (i.e., there were 2 Spanish negative words, 2 Spanish neutral words, 2 Catalan negative words and 2 Catalan neutral words).

Words appeared continuously, remaining on the screen for 2 s with a gap of a further 2 s between each one. They were presented in the middle of the screen, lowercase, in black color against a white background. The font was Arial 40, and the screen resolution was set to 1680 × 1050. Words appeared completely at random, with a different order for each participant. The only exception was with the eight filler items, which appeared in the same order for all participants. Words were presented using SR Research Ltd’s Experiment Builder software.

Immediately after the acquisition phase, participants performed a distractor phase. They watched an animated movie in silence which lasted 5 min and 27 s. Immediately after the end of the movie, participants did the test phase.

**FIGURE 1:**
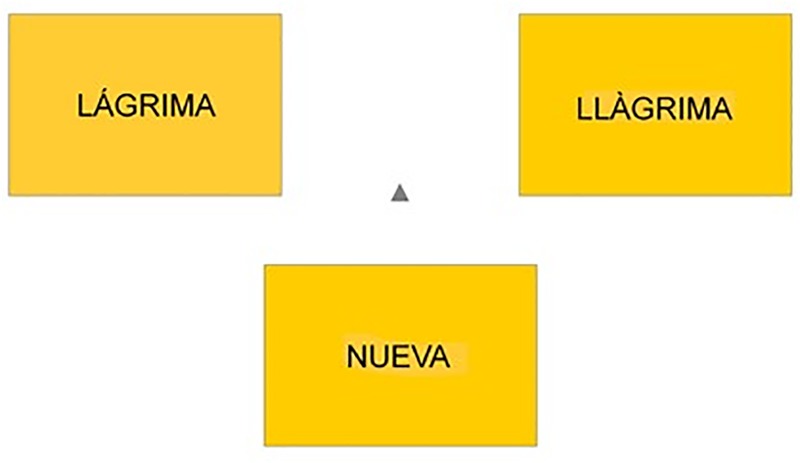
Screenshot of a trial in the test phase. Both words in the upper two boxes mean ‘tear,’ the Spanish word on the left and the Catalan word on the right. The box below contains the Spanish word for ‘new.’

In the test phase there were 120 trials, 60 involving old words (i.e., the critical words that had been presented during the acquisition phase) and 60 involving new words. Like the old words, half of the new words were negative and half were neutral words. Filler items belonging to the primacy and recency portions of the encoding list were not presented during the test phase. There was a single file, which was presented to all the participants. In each trial, a screen with three options was displayed. One option consisted of a Spanish word, and the other option was its Catalan translation. The Spanish word always appeared on the left side of the screen, and the Catalan word always appeared on the right side of the screen (see Figure 1). The third option was the word *NUEVA* (‘new’ in Spanish). Participants had to select from the three options with the mouse, to indicate whether the word had appeared in either Catalan or Spanish during the acquisition phase (i.e., it was an ‘old’ word) or whether it had not appeared (in this case, they had to select the option ‘new’). Words were presented in Arial 24, uppercase, to prevent any strategy based on the recall of the visual form of the words (note that words had been presented in lowercase during the acquisition phase). There was no time limit to respond. After participants’ response, there was an inter-trial interval of 500 ms. Stimuli were presented completely randomly with a different random order created for each participant. Words were presented and data were recorded using SR Research Ltd’s Experiment Builder software.

After the main experimental task, participants were asked to fulfill an online language history questionnaire. The overall experimental session lasted approximately 30 min.

### Results and Discussion

Across the three experiments, we first report item memory scores: hit rate, false alarm rate, A′ and $B_{D}^{\prime \prime }$. Hit rate is the proportion of studied items labeled old without regard to source accuracy (i.e., regardless of language). False alarm rate is the proportion of new items wrongly labeled old. A′ is an index of discriminability which varies from 0 to 1 with 0.5 indicating chance performance. $B_{D}^{\prime \prime }$ is the corresponding measure of bias. Values greater than zero indicate conservative bias, and values less than zero indicate liberal bias (see Macmillan and Creelman, 2005). Subsequently, we report source memory performance. To obtain this, we calculated the unbiased hit rate (Wagner, 1993; Ventura-Bort et al., 2017). The Hit unbiased index (Hu) allows adjusting for response biases, because it not only takes into consideration the hit rates, but also the number of times a response has been given. It is defined as the conjoint probability of the correct identification of a stimulus and the correct use of a response (Wagner, 1993). For instance, the source memory score (Hu) for Spanish negative words was calculated as follows:

$$\frac{SpaNeg\_Lang\_correct}{SpaNeg\_Lang\_correct+SpaNeg\_Lang\_incorrect}*\frac{SpaNeg\_Lang\_correct}{SpaNeg\_Responses}$$

**SpaNeg_Lang_correct**: Number of Spanish negative words correctly labeled as old whose language of presentation was correctly identified.

**SpaNeg_Lang_incorrect**: Number of Spanish negative words correctly labeled as old whose language of presentation was not correctly identified (i.e., participants identified the Catalan word as the presented one).

**SpaNeg_Responses**: Number of times that the identified language for a correctly labeled old word was Spanish (this includes both “SpaNeg_Lang_correct” responses and the number of times that Spanish was incorrectly identified as the language of presentation of a Catalan negative word).

**FIGURE 2:**
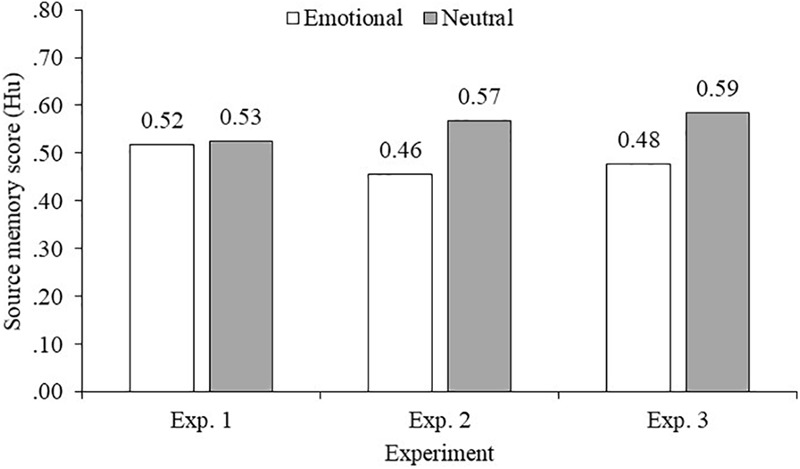
Source memory scores (Hu) collapsed by language for emotional and neutral words in the three experiments.

In all cases, we carried out a 2 (Language) × 2 (Emotion) repeated measures ANOVA. Pairwise Bonferroni corrected comparisons were applied when the ANOVA yielded significant results. Item recognition scores and source memory scores are summarized in Table 2. Source memory scores collapsed by language in the three experiments are displayed in Figure 2.

**Table 2:** Mean, standard deviation (in parentheses), minimum and maximum values (in square brackets) of the dependent variables across experimental conditions in Experiment 1.

Conditions	Hit rate	False alarm rate	A′	$B_{D}^{”}$	Hu
Spanish negative	0.75 (0.17) [0.33 to 1]	0.41 (0.25) [0 to 0.93]	0.74 (0.18) [0.26 to 0.98]	-0.29 (0.56) [-1 to 1]	0.53 (0.18) [0.23 to 1]
Spanish neutral	0.70 (0.20) [0.20 to 1]	0.30 (0.24) [0 to 0.93]	0.77 (0.16) [0.43 to 0.97]	0.01 (0.63) [-1 to 1]	0.54 (0.22) [0.15 to 1]
Catalan negative	0.78 (0.22) [0.13 to 1]	0.36 (0.22) [0 to 0.87]	0.80 (0.11) [0.50 to 0.95]	-0.31 (0.66) [-1 to 1]	0.51 (0.21) [0 to 1]
Catalan neutral	0.70 (0.22) [0.13 to 1]	0.32 (0.24) [0 to 0.93]	0.76 (0.16) [0.36 to 0.98]	-0.06 (0.73) [-1 to 1]	0.51 (0.24) [0.07 to 1]

The ANOVA revealed a main effect of emotion on hit rate, *F*(1,29) = 7.84, *p* = 0.009, $\eta_{p}^{2}$ = 0.21, *MSE* = 0.02, as well as on false alarm rate, *F*(1,29) = 11.55, *p* = 0.002, $\eta_{p}^{2}$ = 0.29, *MSE* = 0.01, where the proportion of hits and false alarms was higher for negative words (*M* = 0.77, *SD* = 0.17, and *M* = 0.39, *SD* = 0.20, for hits and false alarm rates, respectively) than for neutral words (*M* = 0.70, *SD* = 0.19 and *M* = 0.31, *SD* = 0.20). There was no effect of emotion in the A′ parameter, *F*(1,29) = 0.13, *p* = 0.725, $\eta_{p}^{2}$ = 0.004, *MSE* = 0.01. In contrast, the analyses showed an emotion effect in $B_{D}^{”}$, *F*(1,29) = 15.87, *p* < 0.001, $\eta_{p}^{2}$ = 0.35, *MSE* = 0.13, revealing a more liberal criterion for negative words (*M* = -0.30, *SD* = 0.55) than for neutral words (*M* = -0.03, *SD* = 0.55). Neither language nor the interaction of emotion by language was significant in any of the analyses (all *p*s > 0.095).

The analysis of the source memory score (Hu) failed to show a significant effect of any of the factors or interactions involved (all *p*s > 0.314).

The results of this experiment demonstrated that emotion did not affect item memory. Although negative words showed a higher hit rate than neutral words, they also showed a higher false alarm rate. In fact, the false alarm rate was quite high in general in this experiment, suggesting that participants did not discriminate very well between old and new items. A possible reason for that might be that, due to our interest to match the old and new words as best as possible, there is a meaning similarity between the items of both sets. Apart from that, as above mentioned, false alarm rate was higher for negative words than for neutral words, leading discriminability (A′) to be the same in these two types of words. Furthermore, the criterion was more liberal for negative words than for neutral ones. This pattern of findings is in line with previous memory recognition studies (e.g., Doerksen and Shimamura, 2001; Pesta et al., 2001; Comblain et al., 2004; D’Argembeau and Van der Linden, 2004; Mackenzie et al., 2015), suggesting that participants have a tendency to label emotional items as old regardless of whether these had been presented during acquisition or not. However, the present results are at odds with past reports of an enhancing emotion effect in memory (e.g., D’Argembeau and Van der Linden, 2005; Cook et al., 2007; Schmidt et al., 2011; Rimmele et al., 2012; Bisby and Burgess, 2014; Meyer et al., 2015). It should be noted here that such an enhancing effect is much more common in free recall tasks (e.g., Doerksen and Shimamura, 2001; D’Argembeau and Van der Linden, 2004; Davidson et al., 2006; Maddock and Frein, 2009; Nashiro and Mather, 2011; Wang and Fu, 2011; Otani et al., 2012) than in recognition tasks, and in studies involving long retention intervals between the encoding and test phases (Kleinsmith and Kaplan, 1963; see Yonelinas and Ritchey, 2015; Dolcos et al., 2017, for overviews). Moreover, focusing on recognition tasks, the enhancing effect has been reported much more frequently with images (e.g., D’Argembeau and Van der Linden, 2005; Schmidt et al., 2011; Rimmele et al., 2012; Bisby and Burgess, 2014; Meyer et al., 2015) than with words (e.g., Doerksen and Shimamura, 2001; D’Argembeau and Van der Linden, 2004; Davidson et al., 2006). A possible reason for the above findings is that the higher similarity in meaning between emotional words in comparison to neutral words (Talmi and Moscovitch, 2004) benefits free recall but impairs recognition memory, making it more difficult to distinguish between old and new emotional words than between old and new neutral words (White et al., 2014). Such a difficulty might be reduced with images, because they are probably more distinctive than words. On the other hand, long retention intervals would allow an emotion advantage on item memory to emerge due to consolidation processes (LaBar and Cabeza, 2006). The use of an immediate recognition phase, as in the present study, involves testing memory before such consolidation processes have occurred.

A more relevant finding for the purpose of this study is that we failed to find any effect of emotion on source memory, in line with some past studies (Sharot and Phelps, 2004; Sharot and Yonelinas, 2008; Mather et al., 2009; however, see section “Introduction” for a review of the studies finding such an effect). This pattern of findings is not consistent with the proposals of Mather and her co-workers (Mather, 2007; Mather and Sutherland, 2011), considering that language is an intrinsic feature of words which is goal-relevant for the participants during encoding (i.e., they had to encode both the words and the language of presentation). Bearing these two factors in mind, an enhancement should have been clearly observed.

A possible reason for the lack of effects of emotion on source memory might be the nature of the encoding task. During encoding, participants were told that their memory would be tested afterward. It might be that the intention to learn both the words and the language in which they were presented overrides any effect that emotion could have had on source memory (i.e., by increasing performance with both neutral and emotional words). Hence, if we assume that the influence of emotion on cognition may involve automatic processes (e.g., an automatic activation of attention; D’Argembeau and Van der Linden, 2004), it is possible that the effects of emotion on source memory are more easily observed when participants do not intend to memorize the information. In fact, this was what D’Argembeau and Van der Linden (2004) found in a study on source memory for color in which incidental and intentional source encoding were compared. In order to further explore this possibility, we conducted Experiment 2, in which participants were not asked to memorize either the words or the language of presentation, although source information was still goal-relevant, as participants were required to name the words in the correct language.

## Experiment 2

This experiment was carried out in accordance with the code of ethics of the World Medical Association (Declaration of Helsinki) and was approved by the Ethics Committee for Human Research (SECH 017/2015) of the Research Center on Psychology (CIPsi) at the University of Minho. Furthermore, participants gave written informed consent.

### Methods

#### Participants

A sample of 30 undergraduate students (mean age 21.37, *SD* = 4.31, 28 females) from the University Rovira i Virgili (Tarragona, Spain) participated in the experiment either voluntarily or in exchange of academic credits. They were drawn from the same population as those in Experiment 1. However, none of the participants of Experiment 2 had taken part in Experiment 1. All the participants had normal vision or corrected-to- normal vision.

Participants completed the same language history questionnaire as in experiment 1. It revealed that they were highly proficient in Catalan (*M* = 6.77; *SD* = 0.44, Minimum = 5.00, Maximum = 7.00, Range = 2.00) as well as in Spanish (*M* = 6.70; *SD* = 0.49, Minimum = 5.25, Maximum = 7.00, Range = 1.75). The difference between the two languages was not significant; *t*(29) = 0.62, *p* = 0.541, indicating that they were balanced bilinguals. Concerning ratings of frequency and preference of use, although they were close to the mid-point of the 1 to 7 scale (1 = ‘only Catalan,’ 7 = ‘only Spanish’), they were slightly biased toward both using and preferring Catalan over Spanish (*M* = 3.63, *SD* = 1.03, Minimum = 2.00, Maximum = 6.50, Range = 4.50, for frequency of use, and *M* = 3.51, *SD* = 1.32, Minimum = 1.00, Maximum = 5.75, Range = 4.75, for preference of use, respectively), although one-sampled *t*-tests revealed that the difference with respect to the mid-point of the scale was only marginally significant (*p* = 0.061 and *p* = 0.050 for frequency and preference, respectively).

#### Design and Materials

The experimental design and the stimuli for the acquisition phase, as well as for the test phase, were the same as in Experiment 1 (see Table 1).

#### Procedure

The overall experimental session was similar to that in Experiment 1. The distractor phase and the test phase were exactly the same as in that experiment. The difference was seen in the acquisition phase. Participants were told that a series of Spanish and Catalan words would appear on the screen and that they would have to read aloud each word in the correct language as soon as it was presented. We used a naming task in order to make sure that participants were paying attention to the language of the words. They were not informed that their memory was going to be tested afterward. Therefore, they carried out an incidental acquisition phase.

Participants were seated individually in a quiet room with a computer and a microphone. First of all, participants did a practice block, where they had to name a set of digits (from 1 to 10). The purpose of this was to familiarize them with the task. After that, 68 words (i.e., the 60 critical items plus the 8 filler items in the primacy and recency portions of the list) were presented on the screen, one at a time, and the participants’ task was to read them. The sequence of events was as follows: first, a fixation point (‘+’) appeared on the center of the screen for 1 s. Then, a word written in black on a white background was presented (Arial 20, lowercase, at a 1024 × 768 resolution). Participants were instructed to read the word aloud into the microphone connected to the computer. As soon as the participant started to read, the word disappeared from the screen. Two seconds later a message appeared indicating that the spacebar should be pressed to continue. Participants were warned that the words would appear randomly in either Spanish or in Catalan.

After the acquisition phase, the experiment continued with the distractor phase and the test phase, following exactly the same structure and procedure as in Experiment 1.

### Results and Discussion

The same measures as in Experiment 1 were collected (i.e., hit rate, false alarm rate, A′, B″_D_, and source memory score, Hu). Of note, we did not consider those cases in which participants had committed an error during the naming task (e.g., reading the word in the incorrect language, or saying a different word), or had repeated the word more than once. The percentage of omitted data was 8.39% (*SD* = 6.06%).

The results of Experiment 2 are summarized in Table 3. As in Experiment 1, an ANOVA with the factors language and emotion was carried out. The ANOVA on hit rate revealed a main effect of emotion, *F*(1,29) = 6.20, *p* = 0.019, $\eta_{p}^{2}$ = 0.18, *MSE* = 0.02, where the proportion of hits was higher for negative words (*M* = 0.72, *SD* = 0.16), than for neutral words (*M* = 0.65, *SD* = 0.19). There was also a main effect of language, *F*(1,29) = 5.89, *p* = 0.022, $\eta_{p}^{2}$ = 0.17, *MSE* = 0.02, as the hit rate was higher for words presented in Catalan during the acquisition phase (*M* = 0.71, *SD* = 0.17) than for words presented in Spanish (*M* = 0.65, *SD* = 0.17). On the other hand, the analysis of false alarm rate revealed a main effect of emotion, *F*(1,29) = 4.42, *p* = 0.044, $\eta_{p}^{2}$ = 0.13, *MSE* = 0.01, as there were more false alarms with negative words (*M* = 0.31, *SD* = 0.17) than with neutral words (*M* = 0.27, *SD* = 0.18). Furthermore, the interaction between emotion and language also reached statistical significance, *F*(1,29) = 7.71, *p* = 0.010, $\eta_{p}^{2}$ = 0.21, *MSE* = 0.01. This interaction revealed that the difference in false alarm rate between negative and neutral words was only significant for Spanish words (*p* = 0.003) but not for Catalan words (*p* = 0.613). A significant interaction between emotion and language also emerged in A′, *F*(1,29) = 4.59, *p* = 0.041, $\eta_{p}^{2}$ = 0.14, *MSE* = 0.01. This interaction showed that the value of A′ in negative words was higher for Catalan words (*M* = 0.82, *SD* = 0.07) than for Spanish words (*M* = 0.75, *SD* = 0.16). The analyses of the $B_{D}^{”}$ parameter, in turn, revealed a significant effect of emotion, *F*(1,29) = 7.52, *p* = 0.010, $\eta_{p}^{2}$ = 0.21, *MSE* = 0.16, indicating that participants showed a more liberal bias with negative words (*M* = -0.05, *SD* = 0.59) than with neutral words (*M* = 0.15, *SD* = 0.57).

**Table 3:** Mean, standard deviation (in parentheses), minimum and maximum values (in square brackets) of the dependent variables across experimental conditions in Experiment 2.

Conditions	Hit rate	False alarm rate	A′	$B_{D}^{”}$	Hu
Spanish negative	0.69 (0.19) [0.20 to 0.93]	0.34 (0.21) [0 to 0.93]	0.75 (0.14) [0.36 to 0.93]	-0.06 (0.61) [-0.98 to 1]	0.41 (0.19) [0.11 to 0.75]
Spanish neutral	0.61 (0.20) [0.23 to 0.93]	0.24 (0.22) [0 to 0.73]	0.78 (0.10) [0.55 to 0.96]	0.28 (0.65) [-0.95 to 1]	0.52 (0.20) [0.22 to 1]
Catalan negative	0.74 (0.18) [0.40 to 1]	0.29 (0.19) [0 to 0.80]	0.82 (0.07) [0.66 to 0.95]	-0.05 (0.67) [-1 to 1]	0.50 (0.15) [0.20 to 0.74]
Catalan neutral	0.68 (0.22) [0.20 to 1]	0.31 (0.20) [0 to 0.80]	0.76 (0.14) [0.38 to 0.95]	0.01 (0.68) [-1 to 1]	0.61 (0.19) [0.17 to 0.1]

With respect to source memory, the ANOVA on the Hu index showed a main effect of both emotion, *F*(1,29) = 6.76, *p* = 0.015, $\eta_{p}^{2}$ = 0.19, *MSE* = 0.06, and language, *F*(1,29) = 14.25, *p* = 0.001, $\eta_{p}^{2}$ = 0.33, *MSE* = 0.02. These results indicated that source memory was better for neutral words (*M* = 0.57, *SD* = 0.17) than for negative words (*M* = 0.46, *SD* = 0.15) (see Figure 2). Moreover, source memory was better for Catalan words (*M* = 0.56, *SD* = 0.13) than for Spanish words (*M* = 0.47, *SD* = 0.14).

The results concerning item memory revealed, as in Experiment 1, that the proportion of hits was higher for negative words than for neutral words, as was the proportion of false alarms (although in this case, only for Spanish words). Similarly, participants exhibited a more liberal criterion with negative words than with neutral words. These results are in line with the above mentioned tendency to label emotional stimuli as old, regardless of whether they had been presented during acquisition or not (Doerksen and Shimamura, 2001; Pesta et al., 2001; Comblain et al., 2004; D’Argembeau and Van der Linden, 2004; Mackenzie et al., 2015). Furthermore, as in Experiment 1, negative words were not better discriminated than neutral words. It should be noted, however, that the interaction obtained here suggests that Catalan words were better discriminated than Spanish words (at least when they had negative content). In fact, there is a clear tendency in favor of the Catalan language in this experiment: there were more hits in Catalan; there were more false alarms for negative than for neutral words in Spanish, but not in Catalan; negative Catalan words were better discriminated than negative Spanish words, and finally, source memory for the language of words was better when they were presented in Catalan than in Spanish.

A possible reason for the superiority of Catalan here is that participants, although being highly balanced, could be considered slightly more dominant in Catalan than in Spanish (see the preference and frequency data in the description of the participants). It might be easier to remember both the words and their language of presentation in the dominant language than in the non-dominant one. In fact, a study using a free recall and a serial recall paradigm has demonstrated that memory is worse in the less fluent language than in the more fluent language (Francis and Baca, 2014). Another possibility is that Catalan words are more distinctive than Spanish words. That is, although participants are exposed to both languages on a daily basis, and even though they seem to be slightly more dominant in Catalan than in Spanish, Spanish words are more frequently found in a written format than Catalan words (i.e., in books, printed press or Internet) in the Catalan-Spanish bilingual environment of Catalonia. This fact might have contributed to the superior source memory for Catalan words, since, as we know, distinctiveness facilitates memory (Francis and Strobach, 2013).

Apart from the language effects, the most relevant finding of this experiment was the impairment in source memory found for negative words. This result is not consistent with the proposals of Mather (2007) and Mather and Sutherland (2011). Focusing on the object based framework (Mather, 2007), an emotional enhancement in source memory for language would be expected, because language has to be considered an intrinsic feature. We obtained just the opposite. However, this is not the first report of an impairment with an intrinsic source. In particular, Maddock and Frein (2009) found a disadvantage for emotional (negative) words in memory for spatial and temporal information. Similarly, Mackenzie et al. (2015) obtained an impairment in memory for the color superimposed over emotional (negative) scenes in comparison to neutral scenes. They considered that, since participants might have segregated the color from the scenes, it could not be concluded that there is an impairment in memory for intrinsic features. The relevance of the present results is that the impairment was observed with a characteristic which is far more difficult to segregate from items, that is, language. Regarding the ABC theory (Mather and Sutherland, 2011), an emotional advantage in source memory should be expected too, because language was a goal-relevant feature for the task employed here (i.e., participants had to name the words in the correct language), as it was in Experiment 1. However, a notable difference between Experiments 1 and 2 is the intentional vs. incidental nature of the tasks. The different patterns of results in these two experiments suggest that this factor may have a modulatory role in source memory effects. This issue will be discussed in depth in Section “General Discussion.”

Before drawing conclusions from the two experiments conducted here, we considered that it was important to test not only negative stimuli but also positive stimuli. Although some studies in the field have found no differences between positive and negative information (e.g., Mather and Nesmith, 2008; Nashiro and Mather, 2011; Mao et al., 2015), an impairment in memory restricted to negative information has been reported in some cases (Cook et al., 2007; Maddock and Frein, 2009; Otani et al., 2012; Mackenzie et al., 2015). One reason for expecting differences between positive and negative stimuli is that positive and negative information can lead to different processing styles. For instance, Pierce and Kensinger (2011) proposed that negative information encourages item-specific encoding, which does not facilitate the formation of associations. In contrast, positive information leads to global processing that could favor associations (Gasper and Clore, 2002). Hence, we decided to assess source memory for positive words in Experiment 3. According to the above considerations, we expected a source memory enhancement for positive words with respect to neutral words, or at least a lack of impairment.

## Experiment 3

### Methods

This experiment was carried out in accordance with the code of ethics of the World Medical Association (Declaration of Helsinki) and was approved by the Ethics Committee for Human Research (SECH 017/2015) of the Research Center on Psychology (CIPsi) at the University of Minho. Furthermore, participants gave written informed consent.

#### Participants

Thirty participants (mean age = 24.33 years, *SD* = 6.72, 20 females) from the University Rovira i Virgili (Tarragona, Spain) took part in the experiment either voluntarily or in exchange of academic credits. They were from the same population as participants in Experiments 1 and 2. However, none of the participants in Experiment 3 had taken part in the previous experiments. All the participants had normal vision or corrected-to-normal vision.

The language history questionnaire revealed that participants were highly proficient in both Catalan (*M* = 6.76, *SD* = 0.39, Minimum = 5.50, Maximum = 7.00, Range = 1.50) and Spanish (*M* = 6.91, *SD* = 0.26, Minimum = 5.75, Maximum = 7.00, Range = 1.25). The lack of significant differences between the two languages, *t*(29) = 1.66, *p* = 0.107, indicated that they were balanced bilinguals. Concerning frequency of use and language use, self-ratings were close to the middle point of the scale (1 = ‘only Catalan’; 7 = ‘only Spanish’), although there was a slight bias toward Spanish (*M* = 4.48, *SD* = 1.14, Minimum = 2.25, Maximum = 7.00, Range = 4.75, and *M* = 4.36, *SD* = 1.17, Minimum = 1.75, Maximum = 7.00, Range = 5.25, for frequency and preference of use, respectively). This bias was significant for frequency, *t*(29) = 2.28, *p* = 0.030, but not for preference (*p* = 0.10).

#### Design and Materials

The experimental design was the same as in Experiments 1 and 2, including language and emotion as factors. The only difference concerned the levels of the factor emotion, which in this experiment referred to positive and neutral words. Therefore, 30 positive Spanish words (and their Catalan translations) substituted the 30 negative words used in Experiments 1 and 2 (see Table 1 and the Appendix). A word was considered as positive (e.g., *obsequio* in Spanish and *obsequi* in Catalan, meaning ‘gift’), if it had a value of valence above 6 in a 9-point scale (1 = ‘very unpleasant,’ 9 = ‘very pleasant’), and a value of arousal above 5 in a 9-point scale (1 = ‘completely calm,’ 9 = ‘completely excited’). Positive words, as well as the values for the relevant variables, were obtained from the same normative databases used in Experiments 1 and 2. Furthermore, the 30 neutral words were the same as in these experiments. Independent samples *t*-tests revealed significant differences between positive and neutral words both in valence, *t*(58) = 15.64, *p* < 0.001, and arousal, *t*(58) = 13.36, *p* < 0.001, also showing that both conditions were well-matched in the remaining variables (all *p*s > 0.116). Moreover, we selected an additional set of 30 positive words to be used as new words during the test phase of the recognition task, these replacing the negative new words included in Experiments 1 and 2. Neutral new words remained the same. The analyses with the new words revealed significant differences between positive and neutral words in valence, *t*(58) = 17.11, *p* < 0.001, as well as in arousal, *t*(58) = 12.73, *p* < 0.001, whereas they were matched in the remaining variables (all *p*s > 0.106). Finally, the comparison between old and new positive words revealed that they were well-matched (all *p*s > 0.12, except for familiarity, *p* = 0.031).

#### Procedure

The experimental procedure was exactly the same as in Experiment 2.

### Results and Discussion

We collected the same measures as in Experiments 1 and 2. The same analyses were also performed. As in Experiment 2, we did not include in the analyses the words which were read more than once or which had errors during the naming task. The percentage of omitted data was 7.50% (*SD* = 5.79%).

Results of Experiment 3 are summarized in Table 4. The ANOVA on hit rate showed a main effect of language, *F*(1,29) = 10.64, *p* = 0.003, $\eta_{p}^{2}$ = 0.27, *MSE* = 0.01, as the proportion of hits was higher for Catalan words (*M* = 0.73, *SD* = 0.15) than for Spanish words (*M* = 0.66, *SD* = 0.15). On the other hand, the analysis of false alarm rate showed a main effect of emotion, *F*(1,29) = 9.16, *p* = 0.005, $\eta_{p}^{2}$ = 0.24, *MSE* = 0.01, where the proportion of false alarms was higher for positive words (*M* = 0.32, *SD* = 0.20) than for neutral words (*M* = 0.26, *SD* = 0.14). A′ revealed a main effect of language, *F*(1,29) = 5.45, *p* = 0.027, $\eta_{p}^{2}$ = 0.16, *MSE* = 0.01, indicating that participants discriminated Catalan words better (*M* = 0.80, *SD* = 0.12) than Spanish words (*M* = 0.75, *SD* = 0.15). The analyses on $B_{D}^{”}$ failed to show any significant effect (all *p*s > 0.060).

**Table 4:** Mean, standard deviation (in parentheses), minimum and maximum values (in square brackets) of the dependent variables across experimental conditions in Experiment 3.

Conditions	Hit rate	False alarm rate	A′	$B_{D}^{”}$	Hu
Spanish positive	0.69 (0.15) [0.31 to 0.93]	0.35 (0.25) [0 to 0.93]	0.73 (0.16) [0.27 to 0.93]	-0.03 (0.60) [-0.95 to 1]	0.46 (0.17) [0.17 to 0.92]
Spanish neutral	0.64 (0.18) [0.27 to 0.93]	0.27 (0.21) [0 to 0.87]	0.76 (0.13) [0.40 to 0.97]	0.21 (0.60) [-0.94 to 1]	0.56 (0.19) [0.27 to 1]
Catalan positive	0.72 (0.20) [0.21 to 1]	0.29 (0.22) [0 to 0.87]	0.78 (0.17) [0.31 to 0.95]	-0.003 (0.67) [-1 to 1]	0.49 (0.20) [0.08 to 0.92]
Catalan neutral	0.74 (0.17) [0.40 to 1]	0.24 (0.15) [0.07 to 0.60]	0.82 (0.11) [0.57 to 0.97]	-0.004 (0.57) [-1 to 0.90]	0.61 (0.17) [0.29 to 1]

Regarding source memory score, the ANOVA showed a main effect of emotion on Hu, *F*(1,29) = 10.17, *p* = 0.003, $\eta_{p}^{2}$ = 0.26, *MSE* = 0.03, where source memory was better for neutral words (*M* = 0.59, *SD* = 0.17) than for positive words (*M* = 0.48, *SD* = 0.17) (see Figure 2). No other main effects or interactions were observed.

Regarding item memory, in this Experiment we observed an effect of emotion on false alarm rate. This effect was not mirrored by a higher hit rate for positive words. The final outcome, as in experiments 1 and 2, was that emotion did not affect item recognition memory (i.e., A′), in line with past findings (Doerksen and Shimamura, 2001; Pesta et al., 2001; Comblain et al., 2004; D’Argembeau and Van der Linden, 2004; Mackenzie et al., 2015). Although in this experiment emotional content did not affect the criterion significantly, the higher number of false alarms observed with positive words provides additional support for the reported tendency to label emotional stimuli as old, regardless of whether these had been presented during acquisition or not.

Another similarity with respect to experiment 2 was the advantage found for Catalan words (in this experiment, this superiority was observed in the proportion of hits and also in the A′ index). In the Discussion of Experiment 2 we advanced two possible mechanisms of the superiority for Catalan words, the first one related to the slightly higher dominance in Catalan of participants of Experiment 2, and the second one related to the higher distinctiveness of written Catalan words. Considering that participants in Experiment 3 were slightly more dominant in Spanish than in Catalan (see preference and frequency ratings in the description of the participants), the superiority for Catalan cannot be explained by the first mechanism. Rather, the second mechanism seems the most plausible.

Concerning source memory, the results were very similar to those of Experiment 2: we found an impairment in source memory for emotional (positive) words with respect to neutral words. Hence, the supposed global processing elicited by positive information (Gasper and Clore, 2002) would not have facilitated the encoding of the language of the words. Our results with positive words contrast with those of Maddock and Frein (2009) and Mackenzie et al. (2015). In both studies, a decrease in source memory was observed with negative stimuli, but not with positive stimuli. Although the cause of such a discrepancy is not clear, it might be related to the kind of source tested. On the other hand, and for the same reasons outlined in the Discussion of Experiment 2, the present results are not consistent with the proposals of Mather and her co-workers. We will address these issues in detail in Section “General Discussion.”

## General Discussion

The aim of the present study was to test the effects of emotion on source memory for language. Language, as an intrinsic component of words, has never been examined before in this field of research. Highly proficient early bilinguals of Catalan and Spanish performed a recognition task in which they were asked about the language of presentation (Catalan or Spanish) of emotional and neutral words. The type of encoding task varied across experiments, being intentional in Experiment 1 and incidental in Experiments 2 and 3, although in all of these language was a relevant feature for the task in hand. When the encoding task was intentional, there was no effect of emotion on source memory for language (Experiment 1). In contrast, when the encoding task was incidental, there was an impairment in source memory for the language of the presentation of emotional words. Such impairment was observed with both negative words (Experiment 2) and positive words (Experiment 3).

The present results are not consistent with the proposals of Mather (2007) and Mather and Sutherland (2011). According to the object-based framework, an enhancement of source memory for language would be expected in emotional words, considering that language is an intrinsic part of the word. It might be argued, however, that language is not a perceptual within-binding feature. We consider that this is unlikely for the following reasons: on the one hand, word form includes information about the language to which the word belongs (e.g., there are single graphemes as well as bigrams or trigrams which are allowed in a particular language but not in others). Hence, language, as being part of a perceptual symbol (i.e., the form of words), can be considered as a perceptual feature. On the other hand, bearing in mind that episodic memory involves the binding of distinct types of information in a single episode (Murray and Kensinger, 2013), it is clear that encoding words involves encoding both their meaning and their form (including language). In support of that, Marian and Fausey (2006) observed that a group of Spanish–English bilinguals were more accurate in remembering academic-type information when the language of encoding and retrieval were the same than when they were different. These findings were a further example of the phenomenon of context-dependent memory and encoding specificity (Tulving and Thomson, 1973), indicating that language was encoded as part of the encoding episode, acting as a cue that guided memory during retrieval (see also Schroeder and Marian, 2014, for further examples).

As reviewed in the introduction, there are more results in the field which are not consistent with the object-based framework. A limitation of that proposal is the ambiguity of the intrinsic-extrinsic distinction. On the one hand, different criteria have been used to classify stimulus features as intrinsic or extrinsic. In particular, whereas some authors have relied on perceptual characteristics (Mather, 2007), others have relied on conceptual features (e.g., Adolphs et al., 2001). On the other hand, regardless of the particular criterion used, some features are difficult to classify. For instance, whereas location has been considered in the literature as an intrinsic feature (e.g., D’Argembeau and Van der Linden, 2004; Maddock and Frein, 2009), it could also be regarded as an extrinsic feature, if we consider that location is often defined by its spatial relationship to other objects. Hence, further research should be conducted to ascertain which kind of criterion (e.g., perceptual characteristics or conceptual characteristics) is the most relevant in order to distinguish intrinsic and extrinsic features and to account for the divergent pattern of findings in the literature.

In relation to the above, it might also be that dimensions other than the intrinsic/extrinsic distinction are relevant. The proposal of Mather and Sutherland (Mather and Sutherland, 2011) goes in this direction, in suggesting that goal-relevance is a key factor in the emotional effects on source memory. Hence, whether emotional arousal will have a detrimental or an enhancing effect on memory for a particular information (regardless of its intrinsic or extrinsic nature) will depend on its priority at a particular moment. For instance, the association between emotional information and non-emotional information (e.g., color or language), would be enhanced or hindered by the arousing content of the former depending on whether the encoding of this association is relevant for the task at hand. At first glance, the results of the present study are not consistent with this theory. If emotional (arousing) content enhances memory for the information that is task-relevant (and which is in the focus of attention), we should have observed an enhancement in source memory for language in Experiment 1, where language was clearly a goal-relevant feature (i.e., participants were asked to memorize it). A similar enhancement should have been found in Experiments 2 and 3, where language was also goal-relevant (although language was not intentionally encoded here, participants were asked to name the words in the correct language, a task that involves focusing on language). It should be noted, however, that a different prediction could also be made: it might be argued that language was more goal-relevant in Experiment 1 than in Experiment 2, as only in the former were participants explicitly asked to memorize it. If this were the case, a source memory enhancement should be expected only in Experiment 1, and not in Experiments 2 and 3. Although this was not the pattern of results here, what is clear is that results of Experiment 1 differ from those of Experiments 2 and 3. Hence, it seems that apart from goal-relevance, a key point is the intentional/incidental nature of the task, since intentional encoding has attenuated the impairing effects of emotion on source memory. Although the intentional/incidental nature of the task can be regarded as similar to the goal-relevant/irrelevant distinction, both dimensions cannot be equated: there can be intentional tasks where source information is not goal-relevant and incidental tasks where source information is goal-relevant.

With regard to the incidental/intentional nature of the task, findings in the literature are mixed: among the studies that have relied on incidental tasks, some have reported an enhancement in source and relational memory for emotional information (e.g., Mather and Nesmith, 2008; Schmidt et al., 2011), others have reported an impairment (e.g., Bisby and Burgess, 2014), and others have found that the direction of the effect depends on the type of feature assessed (Nashiro and Mather, 2011; Rimmele et al., 2012). In a similar way, both beneficial effects (e.g., Doerksen and Shimamura, 2001) and detrimental effects of emotion (e.g., Mackenzie et al., 2015; Mao et al., 2015) have been reported when participants intentionally encoded the source during acquisition. It is difficult to draw solid conclusions from these studies, as they differ in several methodological aspects, among them the type of feature assessed. More informative are those few studies that have compared an incidental and an intentional encoding task with the same materials, such as in D’Argembeau and Van der Linden (2004) and Maddock and Frein (2009). In both studies, the effect of emotion on memory for the location of words was not modulated by the type of encoding. In contrast, this modulation appeared when source memory referred to the color in which words were presented. In that case, the effects of emotion were restricted to the incidental encoding condition (D’Argembeau and Van der Linden, 2004). Taking into account the above pattern of findings, it seems that the type of source assessed can be a determining factor. A possible reason for such influence is that distinct features might involve different types of processing. Maddock and Frein (2009) pointed out that some stimulus features would be processed automatically whereas other features would require effortful processes. If this is true, the intention to learn might interact, in some way, with the nature of the process involved (e.g., automatic or non-automatic), provoking different effects depending on the particular feature involved.

In relation to the above, it is crucial to examine the role of the type of source (and the type of processing) involved. It is possible that some features of the stimuli, but not others, are processed automatically. A way to elucidate whether a particular feature is automatically encoded is to compare an intentional and an incidental encoding task. If performance does not improve when participants intentionally attend to that feature and try to memorize it in comparison to the incidental condition, it might be reasonable to conclude that the processing of such feature is automatic. When we made this comparison with our data on source memory (i.e., the language of the item), we realized that the average performance in the intentional condition (*M* = 0.52, *SD* = 0.19, Experiment 1) was very similar to that observed with the same stimuli in the incidental condition (*M* = 0.51, *SD* = 0.11, Experiment 2). In fact, the two means did not differ statistically: *t*(58) = 0.27, *p* = 0.789. This seems to suggest that language of presentation, at least in highly proficient bilinguals, is processed automatically. If language, like other features of the stimuli, is automatically encoded, emotional content might interfere with this automatic processing in some way, because it would activate other automatic processing that would divert resources from the automatic processing of language (Maddock and Frein, 2009).

### Caveats and Future Research

Before concluding, several limitations of our study should be mentioned as well as possible directions of future research. The first limitation is that we did not use a retention interval long enough to examine consolidation processes. As already discussed, it might be that the lack of emotion effects on item memory are produced by the short interval used. However, concerning the main focus of interest in this research, the few studies that have investigated this issue have failed to find any modulation of emotional effects on source memory by retention interval (Sharot and Yonelinas, 2008; Wang and Fu, 2011). Nevertheless, as the number of studies is so low, it would be desirable to conduct more research addressing this issue with distinct sources (e.g., language, color, position, order, etc.). Another limitation has to do with the population studied, as the majority of participants were women (78% in average, a proportion which is very similar to most studies in the field). Although there is no reason to believe that the effects of emotion on source memory can be modulated by sex, studies involving a larger percentage of men should be carried out, to obtain a more general picture. On the other hand, participants were not completely homogeneous across experiments regarding their language dominance (balanced in Experiment 1, slightly more dominant in Catalan in Experiment 2 and slightly more dominant in Spanish in Experiment 3). This distribution is a reflect of the characteristics of bilinguals in Catalonia: speakers are early bilinguals, highly proficient in both languages, but usually preferring and using one language over the other. It should be noted, however, that the pattern of results concerning the effects of language on memory was the same in Experiments 2 and 3, in spite of the distinct dominance of their participants, and that language did not interact with the effects of emotion on source memory in any of the experiments. Other limitations are related to the items. In particular, the match between the old and new set of items was not perfect (there was a slight difference in familiarity for positive words and in imageability for neutral words). It is unlikely that such differences have affected the findings obtained, as the materials were the same across experiments and the results were only modulated by the type of encoding task, which seems to be the most relevant factor here. However, matching should be improved in future experiments. On the other hand, and in relation to the materials, a caveat might be that we did only examine cognate words. As stated above, the reason was to make the source memory task more difficult. However, results might not be the same with non-cognate words (i.e., translation equivalents which are not similar in form). Related to that, Catalan and Spanish are very close languages, which share a large proportion of cognate words. It would be interesting to compare less similar pairs of languages, in order to test the generalizability of the present findings. Another issue worth to be commented is that, although our results are not consistent with any of the two proposals of Mather and colleagues, the manipulation used in this study does not allow us to refuse them. The reason is that we did not manipulate the type of source (intrinsic vs. extrinsic, goal-relevant vs. goal-irrelevant). Hence, in order to draw strong conclusions concerning those proposals, further experiments should be conducted. Focusing on language, and regarding the intrinsic–extrinsic distinction, source memory for language might be compared to memory for an extrinsic source (e.g., surrounding border color), using the same words. With respect to the goal-relevant/irrelevant distinction, two situations might be compared, one in which language is goal-relevant (as in this study) and another in which it is goal-irrelevant (e.g., an encoding task which includes words in two languages and where participants have to decide if each word belongs to a particular semantic category, a task where language is clearly goal-irrelevant). Even more, it would be interesting to orthogonally manipulate the intrinsic-extrinsic and the goal-relevant/irrelevant distinctions, in order to know which of these dimensions is more relevant in explaining the effects of emotion on source memory.

## Conclusion

We have obtained an impairment in source memory for the language of presentation of negative and positive words in comparison to neutral words. Such impairment, which was only observed when the encoding of the source was incidental, is not consistent with the proposals of Mather and co-workers. Additional studies should be carried out with language as a source where distinct types of words and long retention intervals are examined. Moreover, further research should be conducted to ascertain if the type of source is a critical factor in determining the effects obtained, in relation to the type of processing involved in any particular source.

## Author Contributions

PF, MC, and MG designed the study and developed the hypotheses. MG searched for the experimental materials and ran the statistical analyses. PF had the primary responsibility for drafting the manuscript. All authors revised the manuscript and approved the final version for submission.

## Conflict of Interest Statement

The authors declare that the research was conducted in the absence of any commercial or financial relationships that could be construed as a potential conflict of interest.
