# Supplementary material for: Emotional Content and Source Memory for Language: Impairment in an Incidental Encoding Task
Source: Front Psychol. 2019 Jan 30;10:65. doi: 10.3389/fpsyg.2019.00065 (PMC6363679; doi:10.3389/fpsyg.2019.00065)
Supplement: Supplementary file 1 [file Table_1.DOCX]

**Appendix.** Negative, neutral, and positive critical stimuli used in the three experiments (Spanish and Catalan words with their English translations in alphabetical order of the Spanish words)

| **Negative Words** |  |  |
| --- | --- | --- |
| Spanish Word | Catalan Word | English Translation |
| auxilio | auxili | help |
| bofetada | bufetada | slap |
| castigo | càstig | punishment |
| cicatriz | cicatriu | scar |
| ciclón | cicló | cyclone |
| condena | condemna | sentence |
| conflicto | conflicte | conflict |
| delincuente | delinqüent | offender |
| demonio | dimoni | demon |
| desigualdad | desigualtat | inequality |
| diablo | diable | devil |
| distorsión | distorsió | distortion |
| divorcio | divorci | divorce |
| espanto | espant | terror |
| frustración | frustració | frustration |
| homicidio | homicidi | homicide |
| huracán | huracà | hurricane |
| incapacidad | incapacitat | inability |
| incomodidad | incomoditat | discomfort |
| inseguridad | inseguretat | insecurity |
| intruso | intrús | intruder |
| inundación | inundació | flood |
| manicomio | manicomi | mental hospital |
| martirio | martiri | martyrdom |
| mentira | mentida | lie |
| rapto | rapte | rapture |
| tabaco | tabac | tobacco |
| testamento | testament | will |
| tristeza | tristesa | sadness |
| violación | violació | violation |
|  |  |  |
| **Neutral Words** |  |  |
| Spanish Word | Catalan Word | English Translation |
| actualidad | actualitat | present |
| aluminio | alumini | aluminium |
| ambiente | ambient | ambient |
| asistencia | assistència | assistance |
| azar | atzar | random |
| blusa | brusa | blouse |
| camionero | camioner | truck driver |
| cemento | ciment | cement |
| chaqueta | jaqueta | jacket |
| cliente | client | client |
| comentario | comentari | comment |
| conducto | conducte | conduit |
| flamenco | flamenc | flamingo |
| folleto | fullet | brochure |
| gestión | gestió | management |
| guardián | guardià | guardian |
| ingenuidad | ingenuïtat | naivety |
| maletero | maleter | trunk |
| maquinilla | maquineta | clippers |
| mecanismo | mecanisme | mechanism |
| músculo | múscul | muscle |
| patente | patent | patent |
| pregón | pregó | proclamation |
| producto | producte | product |
| realización | realització | realization |
| redacción | redacció | drafting |
| secretario | secretari | secretary |
| sendero | sender | path |
| tarjeta | targeta | card |
| vestíbulo | vestíbul | lobby |
|  |  |  |
| **Positive Words** |  |  |
| Spanish Word | Catalan Word | English Translation |
| aceptación | acceptació | acceptance |
| admiración | admiració | admiration |
| afecto | afecte | affection |
| afición | afició | hobby |
| agilidad | agilitat | agility |
| comprensión | comprensió | understanding |
| danza | dansa | dance |
| diseñador | dissenyador | designer |
| educación | educació | education |
| equipaje | equipatge | luggage |
| favorito | favorit | favourite |
| felicidad | felicitat | happiness |
| graduación | graduació | graduation |
| inauguración | inauguració | opening |
| innovación | innovació | innovation |
| invitación | invitació | invitation |
| juventud | joventut | youth |
| mago | mag | magician |
| músico | músic | musician |
| naturaleza | naturalesa | nature |
| obsequio | obsequi | gift |
| paraíso | paradís | paradise |
| premio | premi | prize |
| prestigio | prestigi | prestige |
| restaurante | restaurant | restaurant |
| riqueza | riquesa | wealth |
| romanticismo | romanticisme | romanticism |
| sanidad | sanitat | health |
| seducción | seducció | seduction |
| solidaridad | solidaritat | solidarity |
